# Supplementary material for: Heat adaptation of phage T7 under an extended genetic code
Source: Virus Evol. 2021 Dec 1;7(2):veab100. doi: 10.1093/ve/veab100 (PMC8923235; doi:10.1093/ve/veab100)
Supplement: veab100_Supp [file veab100_supp.zip › Sequencing_Analysis.zip!/Sequencing_Analysis/S2/evidence/JC_63.html]

BRESEQ :: Evidence


| New junction evidence | | | | | | | | | | | |
| --- | --- | --- | --- | --- | --- | --- | --- | --- | --- | --- | --- |
|  | | seq id | position | reads (cov) | reads (cov) | score | skew | freq | annotation | gene | product |
| \* | ? | NC\_001604.1 | = 13568 | 61 (0.010) | 36 (0.010) | 16/588 | 48.0 | 43.4% | intergenic (–/–) | *–/–* | –/– |
|  | ? | NC\_001604.1 | 14352 = | 34 (0.010) |  |  |  |  | intergenic (–/–) | *–/–* | –/– |
| Rejected: Coverage evenness skew score above cutoff. | | | | | | | | | | | |

Only 100 of 130 total aligned reads displayed.  

`TGACAGGATTCTTGATGACTTTCCAGACGACTACGAGAAGTTTCGCTGGAGAGTCCCATTCTAATACGACT‑‑CACTAAAGGAGACACACCATGTTCAAACTGATTAAGAAGTTAGGCCAACTGCTGGTTCGTATGTACAACGTGGAAGCCAAGCGACTGAACGATGAGGCTCGTAAAGAGGCCACACAGTCACGCGCTCTGGCGATTCGCTCCAACGAACTGGCTGACAGTGCATCCACTAAAGTTACCGAGGCTGCCCGTGTGGCAAACCAAGCTCAACAGCTTTCCAAATTCTTTGAGTAATCAA‑‑‑‑‑‑‑‑‑‑‑‑‑‑‑‑‑‑‑‑‑‑‑‑‑‑‑‑‑‑‑‑‑‑‑‑‑‑‑‑‑‑‑‑‑‑‑‑‑‑‑‑‑‑‑‑‑‑‑‑‑‑‑‑‑‑‑‑‑‑‑‑‑‑‑‑‑‑‑‑‑‑‑‑‑‑‑‑‑‑‑‑‑‑‑‑‑‑‑‑‑‑‑‑‑‑‑‑‑‑‑‑‑‑‑‑‑‑‑‑‑‑‑‑‑‑‑‑‑‑‑‑‑‑‑‑‑‑‑‑‑‑‑‑‑‑‑‑‑‑‑‑‑‑‑‑‑‑‑‑‑‑‑‑‑‑‑‑‑‑‑‑‑‑‑‑‑‑‑‑‑‑‑‑‑‑‑‑‑‑‑‑‑‑‑‑‑‑‑‑‑‑‑‑‑‑‑‑‑‑‑‑‑‑‑‑‑‑‑‑‑‑‑‑‑‑‑‑‑‑‑‑‑‑‑‑‑‑‑‑‑‑‑‑‑‑‑‑‑‑‑‑‑‑‑‑‑‑‑‑‑‑‑‑‑‑‑‑‑‑‑‑‑‑‑‑‑‑‑‑‑‑‑‑‑‑‑‑‑‑‑‑‑‑‑‑‑‑‑‑‑  >  NC_001604.1/13263‑13568`  
`‑‑‑‑‑‑‑‑‑‑‑‑‑‑‑‑‑‑‑‑‑‑‑‑‑‑‑‑‑‑‑‑‑‑‑‑‑‑‑‑‑‑‑‑‑‑‑‑‑‑‑‑‑‑‑‑‑‑‑‑‑‑‑‑‑‑‑‑‑‑‑‑‑‑‑‑‑‑‑‑‑‑‑‑‑‑‑‑‑‑‑‑‑‑‑‑‑‑‑‑‑‑‑‑‑‑‑‑‑‑‑‑‑‑‑‑‑‑‑‑‑‑‑‑‑‑‑‑‑‑‑‑‑‑‑‑‑‑‑‑‑‑‑‑‑‑‑‑‑‑‑‑‑‑‑‑‑‑‑‑‑‑‑‑‑‑‑‑‑‑‑‑‑‑‑‑‑‑‑‑‑‑‑‑‑‑‑‑‑‑‑‑‑‑‑‑‑‑‑‑‑‑‑‑‑‑‑‑‑‑‑‑‑‑‑‑‑‑‑‑‑‑‑‑‑‑‑‑‑‑‑‑‑‑‑‑‑‑‑‑‑‑‑‑‑‑‑‑‑‑‑‑‑‑‑‑‑‑‑‑‑‑‑‑‑‑‑‑‑‑‑‑‑‑‑‑‑‑‑‑‑‑‑‑‑‑‑‑‑‑‑‑‑‑‑‑‑‑‑‑‑‑aatcaaTATGATCGTTTCTGACATCGAAGCTAACGCCCTCTTAGAGAGCGTCACTAAGTTCCACTGCGGGGTTATCTACGACTACTCCACCGCTGAGTACGTAAGCTACCGTCCGAGTGACTTCGGTGCGTATCTGGATGCGCTGGAAGCCGAGGTTGCACGAGGCGGTCTTATTGTGTTCCACAACGGTCACAAGTATGACGTTCCTGCATTGACCAAACTGGCAAAGTTGCAATTGAACCGAGAGTTCCACCTTCCTCGTGAGAACTGTATTGACACCCTTGTGTTGTCACGTTTGATTCATTCC  >  NC_001604.1/14352‑14652`  

                                                                                                                                                                                                                                                                                                                                                                                                                                                                                                                                                                                                                                   
`TGACAGGATTCTTGATGACTTTCCAGACGACTACGAGAAGTTTCGCTGGAGAGTCCCATTCTAATACGACT‑‑CACTAAAGGAGACACACCATGTTCAAACTGATTAAGAAGTTAGGCCAACTGCTGGTTCGTATGTACAACGTGGAAGCCAAGCGACTGAACGATGAGGCTCGTAAAGAGGCCACACAGTCACGCGCTCTGGCGATTCGCTCCAACGAACTGGCTGACAGTGTATCCACTAAAGTTACCGAGGCTGCCCGTGTGGCAAACCAAGCTCAACAGCTTTCCAAATTCTTTGAGTA                                                                                                                                                                                                                                                                                                                    >  1:141893/1‑301`  
 `GACAGGATTCTTGATGACTTTCCAGACGACTACGAGAAGTTTCGCTGGAGAGTCCCATTCTAATACGACT‑‑CACTAAAGGAGACACACCATGTTCAAACTGATTAAGAAGTTAGGCCAACTGCTGGTTCGTATGTACAGCGTGGAAGCCAAGCGACAGAACGATGAGGCTCGTAAAGAGCCCACACAGTCACGCGCTCTGGCGATTCGCTCCAACGAACTGGCTGACAGTGATTCCACTAAAGCTACCGAGGCTGCCCGTGTGGCAAACCAAGCCCAACAGCATTCAAAAATCATTGAGAAA                                                                                                                                                                                                                                                                                                                   >  1:327961/1‑301`  
 `GACAGGATTCTTGATGACTTTCCAGACGACTACGAGAAGTTTCGCTGGAGAGTCCCATTCTAATACGACT‑‑CACTAAAGGAGACACACCATGCTCAAACTTATTAAGAAGTTAGGCCAACTGCTGGTTCGTATGTACAACGTGGAAGCCAAGCGACTGAACGATGAGGCTCGTAAAGAGGCCACACAGTCACGCGCTCTGGCGATTCGCTCCAACGAACTGGCTGACAGTGCATCCACTAAAGTTACCGAGGCTGCCCGTGTGGCAAACCAAGCTCAACAGCTTTCCAAATTCTTTGAGTAA                                                                                                                                                                                                                                                                                                                   >  1:120343/1‑301`  
 `GACAGGATTCTTGATGACTTTCCAGACGACTACGAGAAGTTTCACTGGAGAGTCCCATTCTAATACGACT‑‑CACTAAAGGAGACACACCATGTTCAAACTGATTAAGAAGTTAGGCCAACTGCTGGTTCGTATGTACAACGTGGAAGCCAAGCGACTGAACGATGAGGCTCGTAAAGAGGCCACACAGTCACGCGCTCTGGCGATTCGCTCCAACGAACTGGCTGACAGTGCATCCACTAAAGTTACCGAGGCTGCCCGTGTGGCAAACCAAGCTCAACAGCTTTCCAAATTCTTTGAGTAA                                                                                                                                                                                                                                                                                                                   >  1:130451/1‑301`  
  `ACAGGATTCTTGATGACTTTCCAGACGACTACGAGAAGTTTCGCTGGAGAGTCCCATTCTAATACGACT‑‑CACTAAAGGAGACACACCATGTTCAAACTGATTAAGAAGTTAGGCCAACTGCTGGTTCGTATGTACAACGTGGAAGCCAAGCGACTGAACGATGAGTCTCGTAAAGAGGCCACACAGTCACGCGCTCTGGCGATTCGCTCCAACGAACTGGCTGACAGTGCATCCACTAAAGTTACCGAGGCTGCCCGTGTGGCAAACCAAGCCCAACAGCTTTCCAAATTCTTTGAGTAAT                                                                                                                                                                                                                                                                                                                  >  1:809511/1‑301`  
   `CAGGATTCTTGATGACTTTCCAGACGACTACGAGAAGTTTCGCTGGAGAGTCCCATTCTAATACGACT‑‑CACTAAAGGAGACACACCATGTTCAAACTGATTAAGAAGTTAGGCCAACTGCTGGTTCGTATGTACAACGTGGAAGCCAAGCGACTGAACGATGAGGCTCGTAAAGAGGCCACACAGTCACGCGCTCTGGCGATTCGCTCCAACGAACTGGCTGACAGTGCATCCACTAAAGTTACCGAGGCTGCCCGTGTGGCAAACCAAGCTCAACAGCTTTCCAAATTCTTTGAGTAATC                                                                                                                                                                                                                                                                                                                 >  1:504692/1‑301`  
   `CAGGATTCTTGATGACTTTCCAGACGACTACGAGAAGTTTCGCTGGAGAGTCCCATTCTAATACGACT‑‑CACTAAAGGAGACACACCATGTTCAAACTGATTAAGAAGTTAGGCCAACTGCTGGTTCGTATGTACAACGTGGAAGCCAAGCGACTGAACGATGAGGCTCGTAAAGAGGCCACACAGTCACGCGCCCTGGCGATTCGCTCCAACGAACTGGCTGACAGTGCATCCACTAAAGTTACCGAGGCTGCCCGTGTGGCAAACCAAACTCAACAGCTTTCACAATTCTTTGACTAATC                                                                                                                                                                                                                                                                                                                 >  1:608517/1‑301`  
    `gGGATTCTTGATGACTTTCCAGACGAATACGAGAAGGTTCGCGGGAGAGTCCCATTCTAATACGACT‑‑AACTAAAGGAGACACACCATGGTCAAACTGATTAAGAAGTTAGGCCAACTGCTGGTTCGTATGTACAACGTGGAAGCCAAGCGACTGAACGATGAGGCTCGTAAAGAGGCCACACAGTCACGCGCTCTGGCGATTCGCTCCAACGAACTGGCTGACAGTGCATCCACTAAAGTTACCGAGGCTGCCCGTGTGGCAAACCAAGCTCAACAGCTTTCCAAATTCTTTGAGTAATCA                                                                                                                                                                                                                                                                                                                <  1:178466/300‑1`  
    `caGATTCTTGATGACTTTCAAGACGACTACGAGAAGTTTCGCTGGAGAGTCCCATTCTAATACGACT‑‑CACTAAAGGAGACACACCATGTTCAAACTGATTAAGAAGTTAGGCCAACTGCTGGTTCGTATGTACAACGTGGAAGCCAAGCGACTGAACGATGAGGCTCGTAAAGAGGCCACACAGTCACGCGCTCTGGCGATTCGCTCCAACGAACTGGCTGACAGTGCATCAACTAAAGTTACCGAGGCTGCCCGTGTGGCAAACCAAGCTCAACAGCTTTCCAAATTCTTTGAGTAATCA                                                                                                                                                                                                                                                                                                                <  1:193850/299‑1`  
    `agacagCTTGATGACTTTCCAGACGACTACGAGAAGTTTCGCTGGAGAGTCCCATTCTAATACGACT‑‑CACTAAAGGAGACACACCATGTTCAAACTGATTAAGAAGTTAGGCCAACTGCTGGTTCGTATGTACAACGTGGAAGCCAAGCGACTGAACGATGAGGCTCGTAAAGAGGCCACACAGTCACGCGCTCTGGCGATTCGCTCCAACGAACTGGCTGACAGTGCATCCACTAAAGTTACCGAGGCTGCCCGTGTGGCAAACCAAGCTCAACAGCTTTCCAAATTCTTTGAGTAATCA                                                                                                                                                                                                                                                                                                                <  1:105013/295‑1`  
    `AGGATTCTTGATGACTTTCCAGACGACTACGAGAAGTTTCGCTGGAGAGTCCCATTCTAATACGACT‑‑CACTAAAGGAGACACACCATGTTCAAACTGATTAAGAAGTTAGGCCAACTGCTGGTTCGTATGTACAACGTGGAAGCCAAGCGACTGAACGATGAGGCTCGTAAAGAGGCCACACAGTCACGCGCTCTGGCGATTCGCTCCAACGAACTGGCTGACAGTGCATCCACTAAAGTTACCGAGGCTGCCCGTGTGGCAAACCAAGCTCAACAGCTTTCCAAATTCTTTGAGTAATCA                                                                                                                                                                                                                                                                                                                <  1:705301/301‑1`  
    `AGGATTCTTGAGGACTTTCCAGCCGACTACGAGAAGTTTCGCTGGAGAGTCCCATTCTAATACGACT‑‑CACTAAAGGAGACACACCATGTTCAAACTGATTAAGAAGTTAGGCCAACTGCTGGTTCGTATGTACAACGTGGAAGCCAAGCGACTGAACGATGAGGCTCGTAAAGAGGCCACACAGTCACGCGCTCTGGCGATTCGCTCCAACGAACTGGCTGACAGTTCATCCACTAAAGTTACCGAGGCTGCCCGTGTGGCAAACCAAGCTCAACAGCTTTCCAAATTCTTTGAGTAATCA                                                                                                                                                                                                                                                                                                                <  1:393282/301‑1`  
    `AGGATTCGTGAGGACTTTACAGACGACTACGAGAAGTTTCGCTGGAGAGTCCCATTCTAATACGACT‑‑CACTAAAGGAGACAAACCATGTTCAAACTGATTAAGAAGTTAGGCCAACTGCTGGTTCGTATGGACAACGTGGAAGCCAAGCGACTGAACGATGAGGCTCGTAAAGAGGCCACACAGTCACGCGCTCTGGCGATTCGCTCCAACGAACTGGCTGACAGTGCATCCACTAAAGTTACCGAGGCTGCCCGTGTGGCAAACCAAGCTCAACAGCTTTCCAAATTCTTTGAGTAATCA                                                                                                                                                                                                                                                                                                                <  1:592071/301‑1`  
     `GGATTCTTGATTACTTTCCAGACGACTACGAGAAGTTTCGCTGGAGAGTCCCATTCTAATACGACT‑‑CACTAAAGGAGACACACCATGTTCAAACTGATTAAGAAGTTAGGCCAACTGCTGGTTCGTATGTACAACGTGGAAGCCAAGCGACTGAACGATGAGGCTCGTAAAGAGGCCACACAGTCACGCGCTCTGGCGATTCGCTCCAACGAACTGGCTGACAGTGCATCCACTAAAGTTACCGAGGCTGCCCGTGTGGCAAACCAAGCTCAACAGCTTTCCAAATTCTTTGAGTAATCAc                                                                                                                                                                                                                                                                                                               >  1:671680/1‑300`  
     `GGATTCTTGATGACTTTCCAGACGACTACGAGAAGTTTCGCTGGAGAGTCCCATTCTAATACGACTCACACTAAAGGAGACACACCATGTTCAAACTGATTAAGAAGTTATGCCAACTGCTGGTTCGTATGTACAACGTGGAAGCCAAGCGACTGAACGATGAGGCTCGTAAAGAGGCCACACAGTCACGCGCTCTGGCGATTCGCTCCAACGAACTGGCTGACAGTGCATCCACTAAAGTTACCGAGGCTGCCCGTGTGGCAAACCAAGCTCAACAGCTTTCCAAATTCTTTGAGTAATa                                                                                                                                                                                                                                                                                                                 >  1:263423/1‑300`  
     `GGATTCTTGATGACTTTCCAGACGACTACGAGAAGTTTCGCTGGAGAGTCCCATTCTAATACGACT‑‑CACTAAAGGAGACACACCATGTTCAAACTGATTAAGAAGTTAGGCCAACTGCTGGTTCGTATGTACAACGTGGAAGCCAAGCGACTGCACGATGAGGCTCGTAAAGAGGCCACACAGTCACGCGCTCTGGCGATTCGCTCCAACGAACTGGATGACAGTGCATCCACTAAAGTTACCGAGGCTGCCCGTGTGGCAAACCAAGCTCAACAGCTTTCCACATTCTTTGAGTAATCAA                                                                                                                                                                                                                                                                                                               >  1:457328/1‑301`  
     `GGATTCTTGATGACTTTCCAGACGACTACGAGAAGTTTCGCTGGAGAGTCCCATTCTAATACGACT‑‑CACTAAAGGAGACACACCATGTTCAAACTGATTAAGAAGTTAGGCCAACTGCTGGTTCGTATGTACAACGTGGAAGCCAAGCGACTGATCGATGAGGCTCGTAAAGAGGCCACACAGTCACGCGCTCTGGCGATTCGCTCCAACGAACTGGCTGACAGTGCATCCACTAAAGTTACCGAGGCTGCCCGTGTGGCAAACCAAGCTCAACAGCTTTCCAAATTCTTTGAGTAATCAA                                                                                                                                                                                                                                                                                                               >  1:716371/1‑301`  
     `GGATTCTTGATGACTTTCCAGACGACTACGAGAAGTTTCGCTGGAGAGTCCCATTCTAATACGACT‑‑CACTAAAGGAGACACACCATGTTCAAACTGATTAAGAAGTTAGGCCAACTGCTGGTTCGTATGTACAACGTGGAAGCCAAGCGACTGAACGATGAGGCTCGTAAAGAGGCCACACAGTCACGCGCTCTGGCTATTCGCTCCAACGAACTGGCTGACAGTGCATCCACTAAAGTTACCGAGGCTGCCCGTGTGGCAAACCAAGCTCAACCGCTTTCCACATTCTTTGAGTAATCAA                                                                                                                                                                                                                                                                                                               >  1:672486/1‑301`  
     `GGATTCTTGATGACTTTCCAGACGACTACGAGAAGTTTCGCTGGAGAGTCCCATTCTAATACGACT‑‑CACTAAAGGAGACACACCATGTTCAAACTGATTAAGAAGTTAGGCCAACTGCTGGTTCGTATGTACAACGTGGAAGCCAAGCGACTGAACGATGAGGCTCGTAAAGAGGCCACACAGTCACGCGCTCTGGCGATTCGCTCCAACGAACTGGCTGACAGTGCATCCACTAAAGTTACCGAGGCTGCCCGTGTGGCAAACCAAGCTCCACAGCTTTCCAAATTCTTTGAGTAATCAA                                                                                                                                                                                                                                                                                                               >  1:132869/1‑301`  
     `GGATTCTTGATGACTTTCCAGACGACTACGAGAAGTTTCGCTGGAGAGTCCCATTCTAATACGACT‑‑CACTAAAGGAGACACACCATGTTCAAACTGATTAAGAAGTTAGGCCAACTGCTGGTTCGTATGTACAACGTGGAAGCCAAGCGACTGAACGATGAGGCTCGTAAAGAGGCCACACAGTCACGCGCTCTGGCGATTCGCTCCAACGAACTGGCTGACAGTGCATCCACTAAAGTTACCGAGGCTGCCCGTGTGGCAAACCAAGCTCAACAGCTTTCCAAATTCTTTGAGTAATCAc                                                                                                                                                                                                                                                                                                               >  1:465880/1‑300`  
     `GGATTCTTGATGACTTTCCAGACGACTACGAGAAGTTTCGCTGGAGAGTCCCATTCTAATACGACT‑‑CACTAAAGGAGACACACCATGTTCAAACTGATTAAGAAGTTAGGCCAACTGCTGGTTCGTATGTACAACGTGGAAGCCAAGCGACTGAACGATGAGGCTCGTAAAGAGGCCACACAGTCACGCGCTCTGGCGATTCGCTCCAACGAACTGGCTGACAGTGCATCCACTAAAGTTACCGAGGCTGCCCGTGTGGCAAACCAAGCTCAACAGCTTTCCAAATTCTTTGAGTAATCAA                                                                                                                                                                                                                                                                                                               >  1:29277/1‑301`  
     `GGATTCTTGATGACTTTCCAGACGACTACGAGAAGTTTCGCTGGAGAGTCCCATTCTAATACGACT‑‑CACTAAAGGAGACACACCATGTTCAAACTGATTAAGAAGTTAGGCCAACTGCTGGTTCGTATGTACAACGTGGAAGCCAAGCGACTGAACGATGAGGCTCGTAAAGAGGCCACACAGTCACGCGCTCTGGCGATTCGCTCCAACGAACTGGCTGACAGTGCATCCACTAAAGTTACCGAGGCTGCCCGTGTGGCAAACCAAGCTCAACAGCTTTCCAAATTCTTTGAGTAATCAA                                                                                                                                                                                                                                                                                                               >  1:521128/1‑301`  
     `GGATTCTTGATGACTTTCCAGACGACTACGAGAAGTTTCGCTGGAGAGTCCCATTCTAATACGACT‑‑CACTAAAGGAGACACACCATGTTCAAACTGATTAAGAAGTTAGGCCAACTGCTGGTTCGTATGTACAACGTGGAAGCCAAGCGACTGAACGATGAGGCTCGTAAAGAGGCCACACAGTCACGCGCTCTGGCGATTCGCTCCAACGAACTGGCTGACAGTGCATCCACTAAAGTTACCGAGGCTGCCCGTGTGGCAAACCAAGCTCAACAGCTTTCCAAATTCTTTGAGTAATCAA                                                                                                                                                                                                                                                                                                               >  1:547797/1‑301`  
     `GGATTCTTGATGACTTTCCAGACGACTACGAGAAGTTTCGCTGGAGAGTCCCATTCTAATACGACT‑‑CACTAAAGGAGACACACCATGTTCAAACTGATTAAGAAGTTAGGCCAACTGCTGGTTCGTATGTACAACGTGGAAGCCAAGCGACTGAACGATGAGGCTCGTAAAGAGGCCACACAGTCACGCGCTCTGGCGATTCGCTCCAACGAACTGGCTGACAGTGCATCCACTAAAGTTACCGAGGCTGCCCGTGTGGCAAACCAAGCTCAACAGCTTTCCAAATTCTTTGAGTAATCAA                                                                                                                                                                                                                                                                                                               >  1:570057/1‑301`  
     `GGATTCTTGATGACTTTCCAGACGACTACGAGAAGTTTCGCTGGAGAGTCCCATTCTAATACGACT‑‑CACTAAAGGAGACACACCATGTTCAAACTGATTAAGAAGTTAGGCCAACTGCTGGTTCGTATGTACAACGTGGAAGCCAAGCGACTGAACGATGAGGCTCGTAAAGAGGCCACACAGTCACGCGCTCTGGCGATTCGCTCCAACGAACTGGCTGACAGTGCATCCACTAAAGTTACCGAGGCTGCCCGTGTGGCAAACCAAGCTCAACAGCTTTCCAAATTCTTTGAGTAATCAA                                                                                                                                                                                                                                                                                                               >  1:92917/1‑301`  
     `GGATTCTTGATGACTTTCCAGACGACTACGAGAAGTTTCGCTGGAGAGTCCCATTCTAATACGACT‑‑CACTAAAGGAGACACACCATGTTCAAACTGATTAAGAAGTTAGGCCAACTGCTGGTTCGTATGTACAACGTGGAAGCCAAGCGACTGAACGATGAGGCTCGTAAAGAGGCCACACAGTCACGCGCTCTGGCGATTCGCTCCAACGAACTGGCTGACAGTGCATCCACTAAAGTTACCGAGGCTGCCCGTGTGGCAAACCAAGCTCAACAGCTTTCCAAATTCTTTGAGTAATCAA                                                                                                                                                                                                                                                                                                               >  1:91239/1‑301`  
     `GGATTCTTGATGACTTTCCAGACGACTACGAGAAGTTTCGCTGGAGAGTCCCATTCTAATACGACT‑‑CACTAAAGGAGACACACCATGTTCAAACTGATTAAGAAGTTAGGCCAACTGCTGGTTCGTATGTACAACGTGGAAGCCAAGCGACTGAACGATGAGGCTCGTAAAGAGGCCACACAGTCACGCGCTCTGGCGATTCGCTCCAACGAACTGGCTGACAGTGCATCCACTAAAGTTACCGAGGCTGCCCGTGTGGCAAACCAAGCTCAACAGCTTTCCAAATTCTTTGAGTAATCAA                                                                                                                                                                                                                                                                                                               >  1:59369/1‑301`  
     `GGATTCTTGATGACTTTCCAGACGACTACGAGAAGTTTCGCTGGAGAGTCCCATTCTAATACGACT‑‑CACTAAAGGAGACACACCATGTTCAAACTGATTAAGAAGTTAGGCCAACTGCTGGTTCGTATGTACAACGTGGAAGCCAAGCGACTGAACGATGAGGCTCGTAAAGAGGCCACACAGTCACGCGCTCTGGCGATTCGCTCCAACGAACTGGCTGACAGTGCATCCACTAAAGTTACCGAGGCTGCCCGTGTGGCAAACCAAGCTCAACAGCTTTCCAAATTCTTTGAGTAATCAA                                                                                                                                                                                                                                                                                                               >  1:280376/1‑301`  
     `GGATTCTTGATGACTTTCCAGACGACTACGAGAAGTTTCGATGGAGAGTCCCATTCTAATACGACT‑‑CACTAAAGGAGACACACCATGTTCAAACTGATTAAGAAGTTAGGCCAACTGCTGGTTCGTATGTACAACGTGGAAGCCAAGCGACTGAACGATGAGGCTCGTAAAGAGGACACACAGTCACGCGCTCTGGCGATTCGCTCCAACGAACTGGATGACAGTGCATCCACTAAAGTTACCGAGGCTGCCCGTGTGGCAAACCAAGCTCAACAGCTTTCCAAATTCTTTGAGTAATCAA                                                                                                                                                                                                                                                                                                               >  1:105964/1‑301`  
      `GATTCTTGATGACTTTCCAGACGACTACGAGAAGTTTCGCTGGATAGTCCCATTCTAATACGACT‑‑CACTAAAGGAGACACACCATGTTCAAACTGATTAAGAAGTTAGGCCAACTGCTGGTTCGTATGTACAACGTGGAAGCCAATCGACTGAACGATGAGGCTCGTAAAGAGGCCACACAGTCACGCGCTCTGGCGATTCGCTCCAACGAACTGGCTGACAGTGCATCCACTAAAGTTACCGAGGCTGCCCGTGTGGCAAACCAAGCTCAACAGCTTTCCAAATTCTTTGAGTAATCAAT                                                                                                                                                                                                                                                                                                              >  1:666542/1‑301`  
      `GATTCTTGATGACTTTCCAGACGACTACGAGAAGTTTCGCTGGAGAGTCCCATTCTAATACGACT‑‑CACTAAAGGAGACACACCATGTTCAAACTGATTAAGAAGTTAGGCCAACTGCTGGTTCGTATGTACAACGTGGAAGCCAAGCGACTGAACGATGAGGCTCGTAAAGAGGCCACACAGTCACGCGCTCTGGCGATTCGCTCCAACGAACTGGCTGACAGTGCATCCACTAAAGTTACCGAGGCTGCCCGTGTGGCAAACCAAGCTCAACAGCTTTCCAAATTCTTTGAGTAATCAAT                                                                                                                                                                                                                                                                                                              >  1:472264/1‑301`  
       `ATTCTTGATGACTTTCCAGACGACTACGAGAAGTTTCGCTGGAGAGTCCCATTCTAATACGACT‑‑CACTAAAGGAGACACACCATGTTCAAACTGATTAAGAAGTTAGGCCAACTGCTGGTTCGTATGTACAACGTGGAAGCCAAGCGACTGAACGATGAGGCTCGTAAAGAGGCCACACAGTCACGCGCTCTGGCGATTCGCTCCAACGAACTGGCTGACAGTGCATCCACTAAAGTTACCGAGGCTGCCCGTGTGGCCAACCAAGCCCAACAGCTTTCCAAATTCTTTTAGTAATCAATA                                                                                                                                                                                                                                                                                                             >  1:719310/1‑301`  
       `ATTCTTGATGACTTTCCAGACGACTACGAGAAGTTTCGCTGGAGACTCCCATTCTAATACGACT‑‑CACTAAAGGAGACACACCATGTTCAAACTGATTAAGAAGTTAGGCCAACTGCTGGTTCGTATGTACAACGTGGAAGCCAAGCGACTGAACGATGAGGCTCGTAAAGAGGCCACACAGTCACGCGCTCTGGCGATTCGCTCCAACGAACTGGATGACAGTGCATCCACTAAAGTTACCGAGGCTGCCCGTGTGGCAAACCAAGCTCAACAGCTTTCCAAATTCTTTGAGTAATCAATA                                                                                                                                                                                                                                                                                                             <  1:100086/301‑1`  
        `TTCTTGATGACTTTCCAGACGACTACGAGAAGTTTCGCTGGAGAGTCCCATTCTAATACGACT‑‑CACTAAAGGAGACACACCATGTTCAAACTGATTAAGAAGTTAGGCCAACTGCTGATTCGTATGTACAACGTGGAAGCCAAGCGACTGAACGATGAGGCTCGTAAAGAGGCCACACAGTCACGCGCTCTGGCGATTCGCTCCAACGAACTGGCTGACAGTGCATCCACTAAAGTTACCGAGGCTGCCCGTGTGGCAAACCAAGCCACACAACTTTCCAAATTCTTTGAGTActaaaaaa                                                                                                                                                                                                                                                                                                            >  1:412492/1‑293`  
         `gagacagGACTTTCCAGACGACTATGAGAAGTTTCGCTGGAGAGTCCCATTCTAATACGACT‑‑CACTAAAGGAGACACACCATGTTCAAACTGATTAAGAAGTTAGGCCAACTGCTGGTTCGTATGTACAACGTGGAAGCCAAGCGACTGAACGATGAGGCTCGTAAAGAGGCCACACAGTCACGCGCTCTGGCGATTCGCTCCAACGAACTGGCTGACAGTGCATCCACTAAAGTTACCGAGGCTGCCCGTGTGGCAAACCAAGCTCAACAGCTTTCCAAATTCTTTGAGTAATCAATAgg                                                                                                                                                                                                                                                                                                           <  1:723609/294‑3`  
         `TCTTGATGACTTTCCAGACGACTACGAGAAGTTTCGCTGGAGAGTCCCATTCTAATACGACT‑‑CACTAAAGGAGACACACCATGTTCAAACTGATTAAGAAGTTAGGCCAACTGCTGGTTCGTATGTACAACGTGGAAGCCAAGCGACTGAACGATGAGGCTCGTAAAGAGGCCACACAGTCACGCGCTCTGGCGATTCGCTCCAACGAACTGGCTGACAGTGCATCCACTGAAGTTACCGAGGCTGCCCGTGTGGCAAACCAAGCTCAACAGCTTTCCAAATTCTTTGAGTAATCAATAgg                                                                                                                                                                                                                                                                                                           <  1:240340/301‑3`  
         `TCTTGATGACTTTCCAGACGACTACGAGAAGTTTCGCTGGAGAGTCCCATTCTAATACGACT‑‑CACTAAAGGAGACACACCATGTTCAAACTGATTAAGAAGTTAGGCCAACTGCTGGTTCGTATGTACAACGTGGAAGCCAAGCGACTGAACGATGAGGCTCGTAAAGAGGCCACACAGTCACGCGCTCTGGCGATTCGCTCCAACGAACTGGCTGACAGTGCATCCACTAAAGTTACCGAGGCTGCCCGTGTGGCAAACCAAGCTCAACAGCTTTCCAAATTCTTTGAGTAATCAATAgg                                                                                                                                                                                                                                                                                                           <  1:763134/301‑3`  
         `TCTTGATGACTTTCCAGACGACTACGAGAAGTTTCGCTGGAGAGTCCCATTCTAATACGACT‑‑CACTAAAGGAGACACACCATGTTCAAACTGATTAAGAAGTTAGGCCAACTGCTGGTTCGTATGTACAACGTGGAAGCCAAGCGACTGAACGATGAGGCTCGTAAAGAGGCAACACAGTCACGCGCTCTGGCGATTCGCTCCAACGAACTGGCTGACAGTGCATCCACTAAAGTTACCGAGGCTGCCCGTGTGGCAAACCAAGCTCAACAGCTTTCCAAATTCTTTGAGTAATCAATAgg                                                                                                                                                                                                                                                                                                           <  1:388861/301‑3`  
         `TCTTGATGACGTTCCAGACGACTACGAGAAGTTTCGCTGGAGAGTCCCATTCTAATACGACT‑‑CACTAAAGGAGACACACCATGTTCAAACTGATTAAGAAGTTAGGCCAACTGCTGGTTCGTATGTACAACGTGGAAGCCAAGCGACTGAACGATGAGGCTCGTAAAGAGGCCACACAGTCACGCGCTCTGGCGATTCGCTCCAACGAACTGTCTGACAGTGCATCCACTAAAGTTACCGAGGCTGCCCGTGTGGCAAACCAAGCTCAACAGCTTTCCAAATTCTTTGAGTAATCAATAgg                                                                                                                                                                                                                                                                                                           <  1:437245/301‑3`  
          `CTTGATGACTTTCCAGACGACTACGAGAAGTTTCGCTGGAGAGTCCCATTCTAATACGACT‑‑CACTAAAGGAGACACACCATGTTCAAACTGATTAAGAAGTTAGGCCAACTGCTGGTTCGTATGTACAACGTGGAAGCCAAGCGACTGAACGATGAGGCTCGTAAAGAGGCCACACAGTCACGTGCTCTGGCGATTCGCTCCAACGAACTGGCTGACAGTGCATCCACTAAAGTTACCGAGGCTGCCCGTGTGGCAAACCAAGCTCAACAGATTTCCAAATTCTTTGAGTAATCAATAggg                                                                                                                                                                                                                                                                                                          >  1:150885/1‑298`  
          `CTTGATGACTTTCCAGACGACTACGAGAAGTTTCGCTGGAGAGTCCCATTCTAATACGACT‑‑CACTAAAGGAGACACACCATGTTCAAACTGATTAAGAAGTTAGGCCAACTGCTGGTTCGTATGTACAACGTGGAAGCCAAGCGACTGAACGATGAGGCTCGTAAAGAGGCCACACAGTCACGCGCTCTGGCGATTCGCTCCAACGAACTGGCTGACAGTGCATCCACTAAAGTTACCGAGGCTGCCCGTGTGGCAAACCAAGCTCAACAGCTTTCCAAATTCTTTGAGTAATCActgtct                                                                                                                                                                                                                                                                                                          >  1:81323/1‑295`  
          `CTTGATGACTTTCCAGACGACTACGAGAAGTTTCGCTGGAGAGTCCCATTCTAATACGACT‑‑CACTAAAGGAGACACACCATGTTCAAACTGATTAAGAAGTTAGGCCAACTGCTGGTTCGTATGTACAACGTGGAAGCCAAGCGACTGAACGATGAGGCTCGTAAAGAGGCCACACAGTCACGCGCTCTGGCGATTCGCTCCAACGAACTGGCTGACAGTGCATCCACTAAAGTTACCGAGGCTGCCCGTGTGGCAAACCAAGCTCAACAGCTTTCCAAATTCTTTGAGTAATCAATAggc                                                                                                                                                                                                                                                                                                          >  1:358856/1‑298`  
          `CTTGATGACTTTACAGACGACTACGAGAAGTTTCGCTGGAGAGTCCCATTCTAATACGACT‑‑CACTAAAGGAGACACACCATGTTCAAACTGATTAAGAAGTTAGGCCAACTGCTGGTTCGTATGTACAACGTGGAAGCCAAGCGACTGAACGATGAGGCTCGTAAAGAGGCCACACAGTCACGCGCTCTGGCGATTCGCTCCAACGAACTGGCTGACAGTGCATCCACTAAAGTTACCGAGGCTGCCCGTGTGGCAAACCAAGCTCAACAGCTTTCCAAATTCTTTGAGTAATCCATATGA                                                                                                                                                                                                                                                                                                          >  1:93996/1‑301`  
           `TTGATGACTTTCCAGACGACTACGAGAAGTTTCGCTGGAGAGTCCCATTCTAATACGACT‑‑CACTAAAGGAGACACACCATGTTCAAACTGATTAAGAAGTTAGGCCAACTGCTGGTTCGTATGTACAACGTGGAAGCCAAGCGACTGAACGATGAGGCTCGTAAAGAGGCCACACAGTCACGCGCTCTGGCGATTCGCTCCAACGAACTGGCTGACAGTGCATCCACTAAAGTTACCGAGGCTGCCCGTGTGGCAAACCAAGCTCAACAGCTTTCCAAATTCTTTGAGTAATCAATAGGAg                                                                                                                                                                                                                                                                                                         <  1:387523/301‑2`  
           `TTGATGACTTTCCAGACGACTACGAGAAGTTTCGCTGGAGAGTCCCATTCTAATACGACT‑‑CACTAAAGGAGACACACCATGTTCAAACTGATTAAGAAGTTAGGCCAACTGCTGGTTCGTATGTACAACGTGGAAGCCAAGCGACTGAACGATGAGGCTCGTAAAGAGGCCACACAGTCACGCGCTCTGGAGATTCGCTCCAACGAACTGGCTGACAGTGCATCCACTAAAGTTACCGAGGCTGCCCGTGTGGCAAACCAAGCTCAACAGCTTTCAAAATTCTTTGAGTAATCAATATGAT                                                                                                                                                                                                                                                                                                         <  1:110902/301‑1`  
           `TTGATGACTTTCCAGACGACTACGAAGAGTTTCGCGGGAGAGTCCCATTCTAATACGACT‑‑CACTAAAGGAGACACACCAAGTTCAAACAGATTAAGAAGTTAGGCCAACTGCTGGTGCGTATGTACAACGTGGAAGCCAAGCGACTGAACGATGAGGCTCGTAAAGAGGCCACACAGTCACGCGCTCTGGCGATTCGCTCCAACGAACTGGCTGACAGTGCATCCACTAAAGTTACCGAGGCTGCCCGTGTGGCAAACCAAGCTCAACAGCTTTCCAAATTCTTTGAGTAATCAATAGGAg                                                                                                                                                                                                                                                                                                         <  1:173309/301‑2`  
             `GATGACTTTCCAGACGACTACGAGAAGTTTCGCTGGAGAGTCCCATTCTAATACGACT‑‑CACTAAAGGAGACACACCATGTTCAAACTGATTAAGAAGTTAGGCCAACTGCTGGTTCGTATGTACAACGTGGAAGCCAAGCGACTGAACGATGAGGCTCGTAAAGAGGCCACACAGTCACGCGCTCTGGCGATTCGCTCCAACGAACTGGCTGACAGTGCATCCACTAAAGTTACCGAGGCTGCCCGTGTGGCAAACCAAGCTCAACAGCTTTCCAAATTCTTTGAGTAATCAATATGATCG                                                                                                                                                                                                                                                                                                       <  1:115697/301‑1`  
                    `TTCCAGACGACTACGAGAAGTTTCGCTGGAGAGTCCCATTCTAATACGACT‑‑CACTAAAGGAGACACACCATGTTCAAACTGATTAAGAAGTTAGGCCAACTGCTGGTTCGTATGTACTACGTGGAAGCCAAGCGACTGAACGATGAGGCTCGTAAAGAGGCCACACAGTCACGCGCTCTGGCGATTCGCTCCAACGAACTGGCTGACAGTGCATCCACTAAAGTTACCGAGGCTGCCCGTGTGGCAAACCAAGCTCAACAGCTTTCCAAATTCTTTGAGTAATCAATATGActgtctcttt                                                                                                                                                                                                                                                                                                >  1:511538/1‑291`  
                    `TTCCAGACGACTACGAGAAGTTTCGCTGGAGAGTCCCATTCTAATACGACT‑‑CACTAAAGGAGACACACCATGTTCAAACTGATTAAGAAGTTAGGCCAACTGCTGGTTCGTATGTACAACGTGGAAGCCAAGCGACTGAACGATGAGGCTCGTAAAGAGGCCACACAGTCACGCGCTCTGGCGATTCGCTCCAACGAACTGGCTGACAGTGCATCCACTAAAGTTACCGAGGCTGCCCGTGTGGCAAACCAATCTCAACAGCTTTCCAAATTCTTTGAGTAATCAATAGGAgctgtctctt                                                                                                                                                                                                                                                                                                >  1:654101/1‑291`  
                    `TTCCAGACGACTACGAGAAGTTTCGCTGGAGAGTCCCATTCTAATACGACT‑‑CACTAAAGGAGACACACCATGTTCAAACTGATTAAGAAGTTAGGCCAACTGCTGGTTCGTATGTACAACGTGGAAGCCAAGCGACTAAACGATGAGGCTCGTAAAGAGGCCACACAGTCACGCGCTCTGGCGATTCGCTCCAACGAACTGGCTGACAGTGCATCCACTAAAGTTACCTAGGCTGCCCGTGTGGCAAACCAAGCTCAACAGCTTTCCAAATTCTTTGAGTAATCAATATGctgtctcttat                                                                                                                                                                                                                                                                                                >  1:340442/1‑290`  
                      `CCAGACGACTACGAGAAGTTTCGCTGGAGAGTCCCATTCTAATACGACT‑‑CACTAAAGGAGACACACCATGTTCAAACTGATTAAGAAGTTAGGCCAACTGCTGGTTCGTATGTACAACGTGGAAGCCAAGCGACTGACCGATGAGGCTCGTAAAGAGGCCACACAGTCACGCGCTCTGGCGATTCGCTCCAACGAACTGGCTGACAGTGCATCCACTAAAGTTACCGAGGCTGCCCGTGTGGCAAACCAAGCTCAACAGCTTTCCAAATTCTTTGAGTAATCAATAggctgtctcttatac                                                                                                                                                                                                                                                                                              >  1:348107/1‑286`  
                      `CCAGACGACTACGAGAAGTTTCGCTGGAGAGTCCCATTCTAATACGACT‑‑CACTAAAGGAGACACACCATGTTCAAACTGATTAAGAAGTTAGGCCAACTGCTGGTTCGTATGTACAACGTGGAAGCCAAGCGACTGAACGATGAGGCTCGTAAAGAGGCCACACAGTAACGCGCTCTGGCGATTCGCTCCAACGAACTGGCTGACAGTGCATCCACTAAAGTTACCGAGGCTGCCCGTTTGGCAAACCAAGCTCAACAGCTTTCCAAATTCTTTGAGTAATCAATATGATCGTTTATGACA                                                                                                                                                                                                                                                                                              >  1:259590/1‑301`  
                            `GACTACGAGAAGTTTCGCTGGAGAGTCCCATTCTAATACGACT‑‑CACTAAAGGAGACACACCATGTTCAAACTGATTAAGAAGTTAGGCCAACTGCTGGTTCGTATGTACAACGTGGAAGCCAAGCGACTGAACGATGAGGCTCGTAAAGAGGCCACACAGTCACGCGCTCTGGCGATTCGCTCCAACGAACTGGCTGACAGTGCATCCACTAAAGTTACCGAGGCTGCCCGTGTGGCAAACCAAGCTCAACAGCTTTCCAAATTCTTTGAGTAATCAATATGATCGTTTCTGACATCGAAG                                                                                                                                                                                                                                                                                        >  1:308719/1‑301`  
                                       `GTTTCGCTGGAGAGTCCCATTCTAATACGACT‑‑CACTAAAGGAGACACACCATGTTCAAACTGATTAAGAAGTTAGGCCAACTGCTGGTTCGTATGTACAACGTGGAAGCCAAGCGACTGAACGATGAGGCTCGTAAAGAGGCCACACAGTCACGCGCTCTGGCGATTCGCTCCAACGAACTGGCTGACAGTGCATCCACTAAAGTTACCGAGGCTGCCCGTGTGGCAAACCAAGCTCAACAGCTTTCCAAATTCTTTGAGTAATCAATAGGAgctgtctcttatacacatctccgagcccc                                                                                                                                                                                                                                                                             >  1:319605/1‑272`  
                                            `GCTGGAGAGTCCCATTCTAATACGACT‑‑CACTAAAGGAGACACACCATGTTCAAACTGATTAAGAAGTTAGGCCAACTGCTGGTTCGTATGTACAACGTGGAAGCCAAGCGACTGAACGATGAGGCTCGTAAAGAGGCCACACAGTCACGCGCTCTGGCGATTCGCTCCAACGAACTGGCTGACAGTGCATCCACTAAAGTTACCGAGGCTGCCCGTGTGGCAAACCAAGCTCAACAGCTTTCCAAATTCTTTGAGTAATCAATATGATCGTTTCTGACATCGAAGCTAACGCCCTCTTAGc                                                                                                                                                                                                                                                                        >  1:153846/1‑300`  
                                             `gtataagagacagTTCTAATACGACT‑‑CACTAAAGGAGACACACCATGTTCAAACTGATTAAGAAGTTAGGCCAACTGCTGGTTCGTATGTACAACGTGGAAGCCAAGCGACTGAACGATGAGGCTCGTAAAGAGGCCACACAGTCACGCGCTCTGGCGATTCGCTCCAACGAACTTGCTGACAGTGCATCCACTAAAGTTACCGAGGCTGCCCGTGTGGCAAACCAAGCTCAACAGCTTTCCAAATTCTTTGAGTAATCAATATGATCGTTTCTGACATCGAAGCTAACGCCCTCTTAGAG                                                                                                                                                                                                                                                                       <  1:486905/288‑1`  
                                               `GGAGAGTCCCATTCTAATACGACT‑‑CACTAAAGGAGACACACCAGGTTCAAACTGATTAAGAAGTTAGGCCAACTGCTGGTTCGTATGTACAACGTGGAAGCCAAGCGACTGAACGATGAGGCTCGTAAAGAGGCCACACAGTCACGCGCTCTGGCGATTCGCTCCAACGAACTGGCTGACAGTGCATCCACTAAAGTTACCGAGGCTGCCCGTGTGGCAAACCAAGCTCAACAGCTTTCCAAATTCTTTGAGTAATCAATATGATCGTTTCTGACATCGAAGCTAACGCCCTCTTAGAGAG                                                                                                                                                                                                                                                                     <  1:828664/301‑1`  
                                                                         `CACTAAAGGAGACACACCATGTTCAAACTGATTAAGAAGTTAGGCCAACTGCTGGTTCGTATGTACAACGTGGAAGCCAAGCGACTGAACGATGAGGCTCGTAAAGAGGCCACACAGTCACGCGCTCTGGCGATTCGCTCCAACGAACTGGCTGACAGTGCATCCACTACAGTTACCGAGGCTGCCCGGTTGGCAAACCAACCTCAACAGCTTTCCAAATTCTTTGATTAATCAATATCATCGTTTCTTACATCAAACCTACCGCCCTCTTcaagctgtcccttatacccatctccaagcc                                                                                                                                                                                                                                             >  1:132379/1‑271`  
                                                                                                                                                                                                                                                                      `GTGGCAAACCAAGCTCAACAGCTTTCCAAATTCTTTGAGTAATCAATATGATCGTTTCTGACATCGAAGCTAACGCCCTCTTAGAGAGCGTCACTAAGTTCCACTGCGGGGTTATCTACGACTACTCCACCGCTGAGTACGTAAGCTACCGTCCGAGTGACTTCGGTGCGTATCTGGATGCGCTGGAAGCCGAGGTTGCACGAGGCGGTCTTATTGTGTTCCACAACGGTCACAAGTATGACGTTCCTGCATTGACCAAACTGGCAAAGTTGCAATTGAACCGAGAGTTCCACCTTCCTCG                                                <  1:660059/301‑1`  
                                                                                                                                                                                                                                                                                             `cggagatgtgtataagagacagATATGATCGTTTCTGACATCGAAGCTAACGCCCTCTTAGAGAGCGTCACTAAGTTCCACTGCGGGGTTATCTACGACTACTCCACCGCTGAGTACGTAAGCTACCGTCCGAGTGACTTCGGTGCGTATCTGGATGCGCTGGAAGCCGAGGTTGCACGAGGCGGTCTTATTGTGTTCCACAACGGTCACAAGTATGACGTTCCTGCATTGACCAAACTGGCAAAGTTGCAATTGAACCGAGAGTTCCACCTTCCTCGTGAGAACTGTATTGACACCCTTG                         <  1:69985/279‑1`  
                                                                                                                                                                                                                                                                                             `TTCCAAATTCTTTGAGTAATCAATATGATCGTTTCTGACATCGAAGCTAACGCCCTCTTAGAGAGCGTCACTAAGTTCCACTGCGGGGTTATCTACGACTACTCCACCGCTGAGTACGTAAGCTACCGTCCGAGTGACTTCGGTGCGTATCTGGATGCGCTGGAAGCCGAGGTTGCACGAGGCGGTCTTATTGTGTTCCACAACGGTCACAAGTATGACGTTCCTGCATTGACCAAACTGGCAAAGTTGCAATTGAACCGAGAGTTCCACCTctgtctcttatacacatctccgagcccac                         >  1:450496/1‑272`  
                                                                                                                                                                                                                                                                                                `agatgtgtataagagacagATATGATCGTTTCTGACATCGAAGCTAACGCCCTCTTAGAGAGCGTCACTAAGTTCCACTGCGGGGTTATCTACGACTACTCCACCGCTGAGTACGTAAGCTACCGTCCGAGTGACTTCGGTGCGTATCTGGATGCGCTGGAAGCCGAGGTTGCACGAGGCGGTCTTATTGTGTTCCACGACGGTCACAAGTATGACGTTCCTGCATTGACCAAACTGGCAAAGTTGCAATTGAACCGAGAGTTCCACCTTCCTCGTGAGAACTGTATTGACACCCTTGTGT                      <  1:614205/282‑1`  
                                                                                                                                                                                                                                                                                                `agatgtgtataagagacagATATGATCGTTTCTGACATCGAAGCTAACGCCCTCTTAGAGAGCGTCACTAAGTTCCACTGCGGGGTTATCTACGACTACTCCACCGCTGAGTACGTAAGCTACCGTCCGAGTGACTTCGGTGCGTATCTGGATGCGCTGGAAGCCGAGGTTGCACGAGGCGGTCTTATTGTGTTCCACAACGGTCACAAGTATGACGTTCCTGCATTGACCAAACTGGCAAAGTTGCAATTGAACCGAGAGTTCCACCTTCCTCGTGAGAACTGTATTGACACCCTTGTGT                      <  1:753012/282‑1`  
                                                                                                                                                                                                                                                                                                              `gacagATATGATCGTTTCTGACATCGAAGCTAACGCCCTCTTAGAGAGCGTCACTAAGTTCCACTGCGGGGTTATCTACGACTACTCCACCGCTGAGTACGTAAGCTACCGTCCGAGTGACTTCGGTGCGTATCTGGATGCGCTGGAAGCCGAGGTTGCACGAGGCGGTCTTATTGTGTTCCACAACGGTCACAAGTATGACGTTCCTGCATTGACCAAACTGGCAAAGTTGCAATTGAACCGAGAGTTCCACCTTCCTCGTGAGAACTGTATTGACACCCTTGTGTTGTCACGTTTGATT        <  1:686737/296‑1`  
                                                                                                                                                                                                                                                                                                              `aacCAATATGATCGTTTCTGACATCGAAGCTAACGCCCTCTTAGAGAGCGTCACTAAGTTCCACTGCGGGGTTATCTACGACTACTCCACCGCTGAGTACGTAAGCTACCGTCCGAGTGACTTCGGTGCGTATCTGGATGCGCTGGAAGCCGAGGTTGCACGAGGCGGTCTTATTGTGTTCCACAACGGTCACAAGTATGACGTTCCTGCATTGACCAAACTGGCAAAGTTGCAATTGAACCGCGAGTTCCACCTTCCTCGTGAGACCTGTCTTGCCACCCTTGTGTTGTCACGTTTGATT        >  1:355519/4‑301`  
                                                                                                                                                                                                                                                                                                              `aacCAATATGATCGTTTCTGACATCGAAGCTAACGCCCTCTTAGAGAGCGTCACTAAGTTCCACTGCGGGGTTATCTACGACTACTCCACCGCTGAGTACGTAAGCTACCGTCCGAGTGACTTCGGTGCGTATCTGGATGCGCTGGAAGCCGAGGTTGCACGAGGCGGTCTTATTGTGTTCCACAACGGTCACAAGTATGACGTTCCTGCATTGACCAAACTGGCAAAGTTGCAATTGAACCGAGAGTTCCACCTTCCTCGTGAGAACTGTATTGACACCCTTGTGTTGTCACGTTTGATT        >  1:586387/4‑301`  
                                                                                                                                                                                                                                                                                                               `acCAATATGATCGTTTCTGACATCGAAGCTAACGCCCTCTTAGAGAGCGTCACTAAGTTCCACTGCGGGGTTATCTACGACTACTCCACCGCTGAGTACGTAAGCTACCGTCCGAGTGACTTCGGTGCGTATCTTGATGCGCTGGAAGCCGAGGTTGCACGAGGCGGTCTTATTGTGTTCCACAACGGTCACAAGTATGACGTTCCTGCATTGACCAAACTGGCAAAGTTGCAATTGAACCGAGAGTTCCACCTTCCTCGTGAGAACTGTATTGACACCCTTGTGTTGctgtctcttatac       >  1:613038/3‑288`  
                                                                                                                                                                                                                                                                                                                `TCAATATGATCGTTTCTGACATCGAAGCTAACGCCCTCTTAGAGAGCGTCACTAAGTTCCACTGCGGGGTTATCTACGACTACTCCACCGCTGAGTACGTAAGCTACCGTCCGAGTGACTTCGGTGCGTATCTGGATGCGCTGGAAGCCGAGGTTGCACGAGGCGGTCTTATTGTGTTCCACAACGGTCACAAGTATGACGTTCCTGCATTGACCAAACTGGAAAAGTTGCAATTGAACCGAGAGTTCCACCTTCCTCGTGAGAACTGTATTGACACCCTTGTGTTGTCACGTTTGATTCt      >  1:497410/1‑300`  
                                                                                                                                                                                                                                                                                                                 `CAATATGATCGTTTCTTACATCGAAGCTAACGCCCTCTTAGAGAGCGTCACTAAGTTCCACTGCGGGGTTATCTACGACTACTCCACCGCTGAGTACGTAAGCTACCGTCCGAGTGACTTCGGTGCGTATCTGGATTCGCTGGAAGCCGAGGTTGCACGAGGCGGTCTTATTGTGTTCCACAACGGTCACAAGTATGACGTTCCTGCATTGACCAAACTGGCAAAGTTGCAATTGAACCGAGAGTTCCACCTTCCTCGTGAGAACTGTATTGACACCCTTGTGTTTTCACtgtctcttata     >  1:824914/1‑290`  
                                                                                                                                                                                                                                                                                                                 `CAATATGATCGTTTCTGACATCGAAGCTAACGCCCTCTTAGAGAGCGTCACTAAGTTCCACTGCGGGGTTATGTACGACTACTCCACCGCTGAGTACGTAAGCTACCGTCCGAGTGACTTCGGTGCGTAGCAGGATGCGCTGGAAGCGGAGGTTGCACGAGGCGGTCTTATTGTGTTCCACAACGGTCACAAGTATGAAGTTCCTGCATTGACAACACTGGCAAAGTTGCAATGGAACCGAGAGTTCCACCTTCCACGTGAGAAATGTATTGACACCCTTGTGTTGTCAAGTTTGGTTCAT     >  1:743085/1‑301`  
                                                                                                                                                                                                                                                                                                                 `CAATATGATCGTTTCTGACATCGAAGCTAACGCCCTCTTAGAGAGCGTCACTAAGTTCCACTGCGGGGTTATCTACGACTACTCCACCGCTGAGTACGTAAGCTACCGTCCGAGTGACTTCGGTGCGTATCTGGATGCGCTGGAAGCCGAGGTTGCACGAGGCGGTCTTATTGTGTTCCACAACGGTCACAAGTATGACGTTCCTGCATTGACCAAACTGGCAAAGTTGCAATTGAACCGAGAGTTCCACCTTCCTCGTGAGAACTGTATTGACctgtctcttatacacatctccgagccc     >  1:514237/1‑274`  
                                                                                                                                                                                                                                                                                                                 `CAATATGATCGTTTCTGACATCGAAGCTAACGCCCTCTTAGAGAGCGTCACTAAGTTCCACTGCGGGGTTATCTACGACTACTCCACCGCTGAGTACGTAAGCTACCGTCCGAGTGACTTCGGTGCGTATCTGGATGCGCTGGAAGCCGAGGTTGCACGAGGCGGTCTTATTGTGTTCCACAACGGTCACAAGTATGACGTTCCTGCATTGACCAAACTGGCAAAGTTGCAATTGAACCGAGAGTTCCACCTTCCTCGTGAGAACTGTATTGACACCCTTGTGTctgtctcttatacacat     >  1:719087/1‑284`  
                                                                                                                                                                                                                                                                                                                 `CAATATGATCGTTTCTGACATCGAAGCTAACGCCCTCTTAGAGAGCGTCACTAAGTTCCACTGCGGGGTTATCTACGACTACTCCACCGCTGAGTACGTAAGCTACCGTCCGAGTGACTTCGGTGCGTATCTGGATGCGCTGGAAGCCGAGGTTGCACGAGGCGGTCTTATTGTGTTCCACAACGGTCACAAGTATGACGTTCCTGCATTGACCAAACTGGCAAAGTTGCAATTGAACCGAGAGTTCCACCTTCCTCGTGAGAACTGTATTGACACCCTTGTGTTGTCACGTTTGATTCtt     >  1:802281/1‑299`  
                                                                                                                                                                                                                                                                                                                 `CAATATGATCGTTTCTGACATCGAAGCTAACGCCCTCTTAGAGAGCGTCACTAAGTTCCACTGCGGGGTTATCTACGACTACTCCACCGCTGAGTACGTAAGCTACCGTCCGAGTGACTTCGGTGCGTATCTGGATGCGCTGGAAGCCGAGGTTGCACGAGGCGGTCTTATTGTGTTCCACAACGGTCACAAGTATGACGTTCCTGCATTGACCAAACTGGCAAAGTTGCAATTGAACCGAGAGTTCCACCTTCCTCGTGAGAACTGTATTGACACCCTTGTGTTGTCACGTTTGATTCAT     >  1:231505/1‑301`  
                                                                                                                                                                                                                                                                                                                 `CAATATGATCGTTTCTGACATCGAAGCTAACGCCCTCTTAGAGAGCGTCACTAAGTTCCACTGCGGGGTTATCTACGACTACTCCACCGCTGAGTACGTAAGCTACCGTCCGAGTGACTTCGGTGCGTATCTGGATGCGCTGGAAGCCGAGGTTGCACGAGGCGGTCTTATTGTGTTCCACAACGGTCACAAGTATGACGTTCCTGCATTGACCAAACTGGC‑AAGTTGCAATTGAACCGAGAGTTCCACCTTCCTCGTGAGAACTGTATTGACACCCTTGTGTctgtctcttatacacatc    >  1:824070/1‑283`  
                                                                                                                                                                                                                                                                                                                 `CAATATGATCGTTTCTGACATCGAAGCTAACGCCCTCTTAGAGAGCGTCACTAAGTTCCACTGCGGGGTTATCTACGACTACTCCACCGCTGAGTACGTAAGCTACCGTCCGAGTGACTTCGGTGCGTATCTGGATGCGCTGGAAGCCGAGGTTGCACGAGGCGGTCTTATTGTGTTCCACAACGGTCACAAGTATGACGTTCCTGCATAGACCAAACTGGCAAAGTTGCAATTGAACCGAGAGTTCCACCTTCCTAGTGAGAACTGTATTGACACCCTTGTGTTGTCACGTTTGATTCAT     >  1:580127/1‑301`  
                                                                                                                                                                                                                                                                                                                 `CAATATGATCGTTTCTGACATCGAAGCTAACGCCCTCTTAGAGAGCGTCACTAAGTTCCACTGCGGGGTTATCTACGACTACTCCACCGCTGAGTACGTAAGCTACCGTCCGAGTGACTTAGGTGCGTATCTGGATGCGCTGGAAGCCGAGGTTGCACGAGGCGGTCTTATTGTGTTCCACAACGGTCACAAGTATGACGTTCCTGCATTGACCAAACTGGCAAAGTTGCAATTGAACCGAGAGTTCCACCTTCCTCGTGAGAACTGTATTGACACCCTTGTGTTGTCACGTTTGATTCAT     >  1:4986/1‑301`  
                                                                                                                                                                                                                                                                                                                 `CAATATGATCGTTTCTGACATCGAAGCTAACGCCCTCTTAGAGAGCGTAACTAAGTTCCACTGCGGGGTTATCTACGACTACTCCACCGCTGAGTACGTAAGCTACCGTCCGAGTGACTTCGGTGCGTATCTGGATGCGCTGGAAGCCGAGGTTGCACGAGGCGGTCTTATTGTGTTCCACAACGGTCACAAGTATGACGTTCCTGCATTGACCAAACTGGCAAAGTTGCAATTGAACCGAGAGTTCCACCTTCCTCGTGAGAACTGTATTGACACCCTTGTGTTGTCACGTTTGATTCAT     >  1:138950/1‑301`  
                                                                                                                                                                                                                                                                                                                 `CAATATGATCGTTTCTGACATCGAAGCTAACGCCATCTTAGAGAGCGTCACTAAGTTCCACTGCGGGGTTATCTACGACTACTCCACCGCTGAGTACGTAAGCTACCGTCCGAGTGACTTCGGTGCGTATCTGGATGCGCTGGAAGCCGAGGTTGCACGAGGCGGTCTTATTGTGTTCCACAACGGTCACAAGTATGACGTTCCTGCATTGCCCAAACTGGCAAAGTTGCAATTGAACCGGGCGTTACACCTTCCTAGTGAGAACTGTATTGACACCCTTGTGTTGTCACGTCTGATTCAT     >  1:253313/1‑301`  
                                                                                                                                                                                                                                                                                                                  `AATATGATCGTTTCTGACATCGAAGCTAACGCCCTCTTAGAGAGCGTCACTAAGTTCCACTGCGGGGTTATCTACGACTACTCCACCGCTGAGTACGTAAGCTACCGTCCGAGTGACTTCGGTGCGTATCTGGATGCGCTGGAAGCCGAGGTTTCACGAGGCGGTCTTATTGTGTTCCACAACGGTCACAAGTATGACGTTCCTGCATTGACCAAACTGGCAAAGTTGCAATTTAACCGAGAGTTCCACCTTCCTCGTGAGAACTGTATTGACACCCTTGTGTTGTCACGTTTGATTCATT    >  1:625693/1‑301`  
                                                                                                                                                                                                                                                                                                                   `ATATGATCTTTTCTGACATCGAAGCTAACGCCCTCTTAGAGAGCGTCACTAAGTTCCACTGCGGGGTTATCTACGACTACTCCACCGCTGAGTACGTAAGCTACCGTCCGAGTGACTTCGGTGCGTATCTGGATGCGCTGGAAGCCGAGGTTGCACGAGGCGGTCTTATTGTGTTCCACAACGGTCACAAGTATGACGTTCCTGCATTGACCAAACTGGCAAAGTTGCAATTGAACCGAGAGTTCCACCTTCCTCGTGAGAACTGTATTGACACCCTTGTGTTGTCACGTTTGATTCATTC   <  1:41611/301‑1`  
                                                                                                                                                                                                                                                                                                                   `ATATGATCGTTTCTGACATCGAAGCTAACGCCCTCTTAGAGAGCGTCACTAAGTTCCACTGCGGGGTTATCTACGACTACTCTACCGCTGAGTACGTAAGCTACCGTCCGAGTGACTTCGGTGCGTATCTGGATGCGCTGGAAGCCGAGGTTGCACGAGGCGGTCTTATTGTGTTCCACAACGGTCACAAGTATGACGTTCCTGCATTGACCAAACTGGCAAAGTTGCAATTGAACCGAGAGTTCCACCTTCCTCGTGAGAACTGTATTGACACCCTTGTGTTGTCACGTTTGATTCATTC   <  1:133105/301‑1`  
                                                                                                                                                                                                                                                                                                                   `ATATGATCGTTTCTGACATCGAAGCTAACGCCCTCTTAGAGAGCGTCACTAAGTTCCACTGCGGGGTTATCTACGACTACTCCACCTCTGAGTACGTAAGCTACCGTCCGAGTGACTTCGGTGCGTATCTGGATGCGCTGGAAGCCGAGGTTGCACGAGGCGGTCTTATTGTGTTCCACAACGGTCACAAGTATGACGTTCCTGCATTGACCAAACTGGCAAAGTTGCAATTGAACCGAGAGTTCCACCTTCCTCGTGAGAACTGTATTGACACCCTTGTGTTGTCACGTTTTATTCTTTt   >  1:540511/1‑300`  
                                                                                                                                                                                                                                                                                                                   `ATATGATCGTTTCTGACATCGAAGCTAACGCCCTCTTAGAGAGCGTCACTAAGTTCCACTGCGGGGTTATCTACGACTACTCCACCGCTGCGTACGTAAGCTACCGTCCGAGTGACTTCGGTGCGTATCTGGATGCGCTGGAAGCCGAGGTTGCACGAGGCGGTCTTATTGTGTTCCACAACGGTCACAAGTATGACGTTCCTGCATTGACCAAACTGGCAAAGTTGCAATTGAACCGAGAGTTCCACCTTCCTCGTGAGAACTGTATTGACACCCTTGTGTTGTCACGTTTGATTCATTC   >  1:517310/1‑301`  
                                                                                                                                                                                                                                                                                                                   `ATATGATCGTTTCTGACATCGAAGCTAACGCCCTCTTAGAGAGCGTCACTAAGTTCCACTGCGGGGTTATCTACGACTACTCCACCGCTGAGTACGTAAGCTACCGTCCGAGTGACTTCGGTGCGTATCTGGATGGGCTGGAAGCCGAGGTTGCACGAGGCGGTCTTATTGTGTTCCACAACGGTCACAAGTATGACGTTCCTGCATTGACCAAACTGGCAAAGTTGAAATTGAAACGGGAGTTCCACCTTCCTCGTGAAAACTGGATTTACCCCCTTGGGTTGTCCCGTTTGATCCATTC   >  1:301562/1‑301`  
                                                                                                                                                                                                                                                                                                                   `ATATGATCGTTTCTGACATCGAAGCTAACGCCCTCTTAGAGAGCGTCACTAAGTTCCACTGCGGGGTTATCTACGACTACTCCACCGCTGAGTACGTAAGCTACCGTCCGAGTGACTTCGGTGCGTATCTGGATGCTCTGGAAGCCGAGGTTGCACGAGGCGGTCTTATTGTGTT‑CACAACGGTCACAAGTATGACGTTCCTGCATTGACCAAACTGGCAAAGTTGCAATTGAACCGAGAGTTCCACCTTCCTCGTGAGAACTGTATTGACACCCTTGTGTTGTCACGTTTGATTCATTCC  <  1:643769/301‑1`  
                                                                                                                                                                                                                                                                                                                   `ATATGATCGTTTCTGACATCGAAGCTAACGCCCTCTTAGAGAGCGTCACTAAGTTCCACTGCGGGGTTATCTACGACTACTCCACCGCTGAGTACGTAAGCTACCGTCCGAGTGACTTCGGTGCGTATCTGGATGCGCTGGAAGCCGGGGTTGCACGAGGCGGTCTTATTGTGTTCCACAACGGTAACAAGTATGACGTTCCTGCATTGACCAAACTGGCAAAGTTGCAATTGAACCGAGAGTTCCACCTTCCTCGTGAGAACTGTATTGACACCCTTGTGTTGTAACGTTTGCTTCATTC   >  1:77429/1‑301`  
                                                                                                                                                                                                                                                                                                                   `ATATGATCGTTTCTGACATCGAAGCTAACGCCCTCTTAGAGAGCGTCACTAAGTTCCACTGCGGGGTTATCTACGACTACTCCACCGCTGAGTACGTAAGCTACCGTCCGAGTGACTTCGGTGCGTATCTGGATGCGCTGGAAGCCGAGTTTGCACGAGGCGGTCTTATTGTGTTCCACAACGGTCACAAGTATGACGTTTCTGCATTGACCAAACTGGCAAAGTTGCAATTGAACCGAGAGTTCCACCTTCCTCGTGAGAACTGAATTGACACCCTTGTGTTGTCACGTTTGATTCATTC   >  1:165689/1‑301`  
                                                                                                                                                                                                                                                                                                                   `ATATGATCGTTTCTGACATCGAAGCTAACGCCCTCTTAGAGAGCGTCACTAAGTTCCACTGCGGGGTTATCTACGACTACTCCACCGCTGAGTACGTAAGCTACCGTCCGAGTGACTTCGGTGCGTATCTGGATGCGCTGGAAGCCGAGGTTGCACGAGTCGGTCTTATTGTGTTCCACAACGGTCACAAGTATGACGTTCCTGCATTGACCAACCTGGCAAAGTTGCAATTGAACCGAGAGTTCCACCTTCCTCGTGAGAACTGTATTGTCACCCTTGTGTTGTCACGTTTGATTCATTt   >  1:27362/1‑300`  
                                                                                                                                                                                                                                                                                                                   `ATATGATCGTTTCTGACATCGAAGCTAACGCCCTCTTAGAGAGCGTCACTAAGTTCCACTGCGGGGTTATCTACGACTACTCCACCGCTGAGTACGTAAGCTACCGTCCGAGTGACTTCGGTGCGTATCTGGATGCGCTGGAAGCCGAGGTTGCACGAGGCGGTCTTATTGTGTTCCACAACGGTCACAAGTATGACGTTCCTGCATTGACCAAACTGGCAAAGTTGCAATTGAACCGAGAGTTCCACCTTCCTCGTGAGAACTGTATTGACACCCTTGTGTTGTCACGctgtctcttata   >  1:411258/1‑289`  
                                                                                                                                                                                                                                                                                                                   `ATATGATCGTTTCTGACATCGAAGCTAACGCCCTCTTAGAGAGCGTCACTAAGTTCCACTGCGGGGTTATCTACGACTACTCCACCGCTGAGTACGTAAGCTACCGTCCGAGTGACTTCGGTGCGTATCTGGATGCGCTGGAAGCCGAGGTTGCACGAGGCGGTCTTATTGTGTTCCACAACGGTCACAAGTATGACGTTCCTGCATTGACCAAACTGGCAAAGTTGCAATTGAACCGAGAGTTCCACCTTCCTCGTGAGAACTGTATTGACACCCTTGTGTTGTCACGTTTGATTCATTC   >  1:756143/1‑301`  
                                                                                                                                                                                                                                                                                                                   `ATATGATCGTTTCTGACATCGAAGCTAACGCCCTCTTAGAGAGCGTCACTAAGTTCCACTGCGGGGTTATCTACGACTACTCCACCGCTGAGTACGTAAGCTACCGTCCGAGTGACTTCGGTGCGTATCTGGATGCGCTGGAAGCCGAGGTTGCACGAGGCGGTCTTATTGTGTTCCACAACGGTCACAAGTATGACGTTCCTGCATTGACCAAACTGGCAAAGTTGCAATTGAACCGAGAGTTCCACCTTCCTCGTGAGAACTGTATTGACACCCTTGTGTTGTCACGTTTGATTCATTC   >  1:737548/1‑301`  
                                                                                                                                                                                                                                                                                                                   `ATATGATCGTTTCTGACATCGAAGCTAACGCCCTCTTAGAGAGCGTCACTAAGTTCCACTGCGGGGTTATCTACGACTACTCCACCGCTGAGTACGTAAGCTACCGTCCGAGTGACTTCGGTGCGTATCTGGATGCGCTGGAAGCCGAGGTTGCACGAGGCGGTCTTATTGTGTTCCACAACGGTCACAAGTATGACGTTCCTGCATTGACCAAACTGGCAAAGTTGCAATTGAACCGAGAGTTCCACCTTCCTCGTGAGAACTGTATTGACACCCTTGTGTTGTCACGTTTGATTCATTC   >  1:638874/1‑301`  
                                                                                                                                                                                                                                                                                                                   `ATATGATCGTTTCTGACATCGAAGCTAACGCCCTCTTAGAGAGCGTCACTAAGTTCCACTGCGGGGTTATCTACGACTACTCCACCGCTGAGTACGTAAGCTACCGTCCGAGTGACTTCGGTGCGTATCTGGATGCGCTGGAAGCCGAGGTTGCACGAGGCGGTCTTATTGTGTTCCACAACGGTCACAAGTATGACGTTCCTGCATTGACCAAACTGGCAAAGTTGCAATTGAACCGAGAGTTCCACCTTCCTCGTGAGAACTGTATTGACACCCTTGTGTTGTCACGTTTGATTCATTC   >  1:636844/1‑301`  
                                                                                                                                                                                                                                                                                                                   `ATATGATCGTTTCTGACATCGAAGCTAACGCCCTCTTAGAGAGCGTCACTAAGTTCCACTGCGGGGTTATCTACGACTACTCCACCGCTGAGTACGTAAGCTACCGTCCGAGTGACTTCGGTGCGTATCTGGATGCGCTGGAAGCCGAGGTTGCACGAGGCGGTCTTATTGTGTTCCACAACGGTCACAAGTATGACGTTCCTGCATTGACCAAACTGGCAAAGTTGCAATTGAACCGAGAGTTCCACCTTCCTCGTGAGAACTGTATTGACACCCTTGTGTTGTCACGTTTGATTCATTC   <  1:585074/301‑1`  
                                                                                                                                                                                                                                                                                                                   `ATATGATCGTTTCTGACATCGAAGCTAACGCCCTCTTAGAGAGCGTCACTAAGTTCCACTGCGGGGTTATCTACGACTACTCCACCGCTGAGTACGTAAGCTACCGTCCGAGTGACTTCGGTGCGTATCTGGATGCGCTGGAAGCCGAGGTTGCACGAGGCGGTCTTATTGTGTTCCACAACGGTCACAAGTATGACGTTCCTGCATTGACCAAACTGGCAAAGTTGCAATTGAACCGAGAGTTCCACCTTCCTCGTGAGAACTGTATTGACACCCTTGTGTTGTCACGTTTGATTCATTC   <  1:536864/301‑1`  
                                                                                                                                                                                                                                                                                                                   `ATATGATCGTTTCTGACATCGAAGCTAACGCCCTCTTAGAGAGCGTCACTAAGTTCCACTGCGGGGTTATCTACGACTACTCCACCGCTGAGTACGTAAGCTACCGTCCGAGTGACTTCGGTGCGTATCTGGATGCGCTGGAAGCCGAGGTTGCACGAGGCGGTCTTATTGTGTTCCACAACGGTCACAAGTATGACGTTCCTGCATTGACCAAACTGGCAAAGTTGCAATTGAACCGAGAGTTCCACCTTCCTCGTGAGAACTGTATTGACACCCTTGTGTTGTCACGTTTGATTCATTC   >  1:431822/1‑301`  
                                                                                                                                                                                                                                                                                                                   `ATATGATCGTTTCTGACATCGAAGCTAACGCCCTCTTAGAGAGCGTCACTAAGTTCCACTACGGGGTTATCTACGACTACTCCACCGCTGAGTACGTAAGCTACCGTCCGAGTGACTTCGGTGCGTCTCTGGCTGCGCTGGAAGCCGAGGTTGCACGAGGCGGTCTTATTGTGTTCCACAACGGTCACAAGTATGACGTTCCTGCATTGACCAAACTGGCAAAGTTGCAATTGAACCGAGAGTTCCACCTTCCTCGTGAGAACTGTATTGACACCCTTGTGTTGTCCCGTTTGATTCATTC   >  1:809544/1‑301`  
                                                                                                                                                                                                                                                                                                                   `ATATGATCGTTTCTGACATCGAAGCTAACGCACTCTTAGAGAGCGTCACTAAGTTCCACTGCGGGGTTATCTACGACTACTCCACCGCTGAGTACGTAAGCTACCGTCCGAGTGACTTCGGTGCGTATCTGGATGCGCTGGAAGCCGAGGTTGCACGAGGCGGTCTTATTGTGTTCCACAACGGTCACAAGTATGACGTTCCTGCATTGACCAAACTGGCAAAGTTGCAATTGAACCGAGAGTTCCACCTTCCTCGTGAGAACTGTATTGACACCCTTGTGTTGTCACGTTTGATTCATTC   >  1:697391/1‑301`  
                                                                                                                                                                                                                                                                                                                   `ATATGAACTTTTCTGACAACGAAGCTAACGCCCTCGTAGAGAGCGTCACTAAGTTCCACTGCGGGGTTATCTACGACTACTCCACCGCTGAGTACGTAAGCTACCGTCCGAGTGACTTCGGTGCGTATCTGGATGCGCTGGAAGCCGAGGTGGCACGAGGCGGTCTTATTGTGTTCCAAAACGGTCACAAGTATGACGTTCCCGCATTGACCAAACTGGCAAAGTTGCAATTGAACCGAGAGTTCCACCTTCCTCGTGAGAACTGTATTGACACCCTTGTGTTGTCACGTTTGATTCATTC   <  1:591058/301‑1`  

                                                                                                                                                                                                                                                                                                                                                                                                                                                                                                                                                                                                                                   
`TGACAGGATTCTTGATGACTTTCCAGACGACTACGAGAAGTTTCGCTGGAGAGTCCCATTCTAATACGACT‑‑CACTAAAGGAGACACACCATGTTCAAACTGATTAAGAAGTTAGGCCAACTGCTGGTTCGTATGTACAACGTGGAAGCCAAGCGACTGAACGATGAGGCTCGTAAAGAGGCCACACAGTCACGCGCTCTGGCGATTCGCTCCAACGAACTGGCTGACAGTGCATCCACTAAAGTTACCGAGGCTGCCCGTGTGGCAAACCAAGCTCAACAGCTTTCCAAATTCTTTGAGTAATCAA‑‑‑‑‑‑‑‑‑‑‑‑‑‑‑‑‑‑‑‑‑‑‑‑‑‑‑‑‑‑‑‑‑‑‑‑‑‑‑‑‑‑‑‑‑‑‑‑‑‑‑‑‑‑‑‑‑‑‑‑‑‑‑‑‑‑‑‑‑‑‑‑‑‑‑‑‑‑‑‑‑‑‑‑‑‑‑‑‑‑‑‑‑‑‑‑‑‑‑‑‑‑‑‑‑‑‑‑‑‑‑‑‑‑‑‑‑‑‑‑‑‑‑‑‑‑‑‑‑‑‑‑‑‑‑‑‑‑‑‑‑‑‑‑‑‑‑‑‑‑‑‑‑‑‑‑‑‑‑‑‑‑‑‑‑‑‑‑‑‑‑‑‑‑‑‑‑‑‑‑‑‑‑‑‑‑‑‑‑‑‑‑‑‑‑‑‑‑‑‑‑‑‑‑‑‑‑‑‑‑‑‑‑‑‑‑‑‑‑‑‑‑‑‑‑‑‑‑‑‑‑‑‑‑‑‑‑‑‑‑‑‑‑‑‑‑‑‑‑‑‑‑‑‑‑‑‑‑‑‑‑‑‑‑‑‑‑‑‑‑‑‑‑‑‑‑‑‑‑‑‑‑‑‑‑‑‑‑‑‑‑‑‑‑‑‑‑‑‑‑‑  >  NC_001604.1/13263‑13568`  
`‑‑‑‑‑‑‑‑‑‑‑‑‑‑‑‑‑‑‑‑‑‑‑‑‑‑‑‑‑‑‑‑‑‑‑‑‑‑‑‑‑‑‑‑‑‑‑‑‑‑‑‑‑‑‑‑‑‑‑‑‑‑‑‑‑‑‑‑‑‑‑‑‑‑‑‑‑‑‑‑‑‑‑‑‑‑‑‑‑‑‑‑‑‑‑‑‑‑‑‑‑‑‑‑‑‑‑‑‑‑‑‑‑‑‑‑‑‑‑‑‑‑‑‑‑‑‑‑‑‑‑‑‑‑‑‑‑‑‑‑‑‑‑‑‑‑‑‑‑‑‑‑‑‑‑‑‑‑‑‑‑‑‑‑‑‑‑‑‑‑‑‑‑‑‑‑‑‑‑‑‑‑‑‑‑‑‑‑‑‑‑‑‑‑‑‑‑‑‑‑‑‑‑‑‑‑‑‑‑‑‑‑‑‑‑‑‑‑‑‑‑‑‑‑‑‑‑‑‑‑‑‑‑‑‑‑‑‑‑‑‑‑‑‑‑‑‑‑‑‑‑‑‑‑‑‑‑‑‑‑‑‑‑‑‑‑‑‑‑‑‑‑‑‑‑‑‑‑‑‑‑‑‑‑‑‑‑‑‑‑‑‑‑‑‑‑‑‑‑‑‑‑aatcaaTATGATCGTTTCTGACATCGAAGCTAACGCCCTCTTAGAGAGCGTCACTAAGTTCCACTGCGGGGTTATCTACGACTACTCCACCGCTGAGTACGTAAGCTACCGTCCGAGTGACTTCGGTGCGTATCTGGATGCGCTGGAAGCCGAGGTTGCACGAGGCGGTCTTATTGTGTTCCACAACGGTCACAAGTATGACGTTCCTGCATTGACCAAACTGGCAAAGTTGCAATTGAACCGAGAGTTCCACCTTCCTCGTGAGAACTGTATTGACACCCTTGTGTTGTCACGTTTGATTCATTCC  >  NC_001604.1/14352‑14652`

  

Alignment Legend

Aligned base mismatch/match (shaded by quality score): `ATCG/ATCG` `< 3 ≤` `ATCG/ATCG` `< 16 ≤` `ATCG/ATCG` `< 27 ≤` `ATCG/ATCG` `< 37 ≤` `ATCG/ATCG` `< 38 ≤` `ATCG/ATCG`

Unaligned base: `atcg`    Masked matching base: `atcg`    Alignment gap: `‑`    Deleted base: `‑`

Reads not counted as support for junction

read\_name Not counted due to insufficient overlap past the breakpoint.

read\_name Not counted due to not crossing MOB target site duplication.
